# Agavins Increase Neurotrophic Factors and Decrease Oxidative Stress in the Brains of High-Fat Diet-Induced Obese Mice

**DOI:** 10.3390/molecules21080998

**Published:** 2016-08-02

**Authors:** Elena Franco-Robles, Mercedes G. López

**Affiliations:** Departamento de Biotecnología y Bioquímica, Centro de Investigación y de Estudios Avanzados del IPN, Unidad de Biotecnología e Ingeniería Genética de Plantas, Irapuato, Guanajuato C.P. 36821, México; efranco@ira.cinvestav.mx

**Keywords:** agavins, high-fat diet, brain, lipids, oxidative stress

## Abstract

Background: Fructans obtained from agave, called agavins, have recently shown significant benefits for human health including obesity. Therefore, we evaluated the potential of agavins as neuroprotectors and antioxidants by determining their effect on brain-derived neurotrophic factor (BDNF) and glial-derived neurotrophic factor (GDNF) as well as oxidative brain damage in of obese mice. Methods: Male C57BL/6J mice were fed a high-fat diet (HFD) and treated daily with 5% (HFD/A5) or 10% (HFD/A10) of agavins or a standard diet (SD) for 10 weeks. The levels of BDNF and GDNF were evaluated by ELISA. The oxidative stress was evaluated by lipid peroxidation (TBARS) and carbonyls. SCFAs were also measured with GC-FID. Differences between groups were assessed using ANOVA and by Tukey’s test considering *p* < 0.05. Results: The body weight gain and food intake of mice HFD/A10 group were significantly lower than those in the HFD group. Agavins restored BDNF levels in HFD/A5 group and GDNF levels of HFD/A5 and HFD/A10 groups in cerebellum. Interestingly, agavins decreased TBARS levels in HFD/A5 and HFD/A10 groups in the hippocampus, frontal cortex and cerebellum. Carbonyl levels were also lower in HFD/A5 and HFD/A10 for only the hippocampus and cerebellum. It was also found that agavins enhanced SCFAs production in feces. Conclusion: Agavins may act as bioactive ingredients with antioxidant and protective roles in the brain.

## 1. Introduction

Obesity is currently a public health problem worldwide. This problem, which nowadays can be called an epidemic, is caused by various factors such as the intake of foods with high caloric content coupled with physical inactivity and genetics, which leads to other diseases, particularly type 2 diabetes and cardio-vascular disease [1,2]. Recently, some carbohydrates have been of particular interest in the field of obesity because they are involved in lipid and carbohydrate metabolism, as in the case of fructans including agave fructans coming from *Agave* plants from Mexico [3]. Agave fructans are non-reducing carbohydrates formed by a complex mixture. They present a highly branched structure that includes β(2→1) and β(2→6) linkages and are called agavins [4]. Importantly, it has been found that differences in the effect of prebiotics may be due to the molecular conformation, degree of polymerization and the species of each fructan [5,6,7]. Fructans are resistant to hydrolysis by all human digestive enzymes but are fermented by the intestinal microbiota forming short-chain fatty acids (SCFAs), which are known to have important implications in host health [8]. Moreover, fructans may also act as scavengers of reactive oxygen species [9], decreasing inflammation and improving redox status. However, the roles of fructans in the brain have been unexplored until now. In this study, we focused on the hippocampus, frontal cortex and cerebellum because they are cortical areas particularly susceptible to diet-induced impairment, especially the hippocampus as it is an area involved in learning and memory [10,11,12,13].

Elevated intake of saturated fat and simple sugars increase incidence of cognition disruption and neurodegenerative diseases including Alzheimer’s disease [10,11], as well as impairing hippocampal synaptic plasticity and cognitive abilities such as learning and memory through brain derived neurotrophic factor (BDNF) [12,13,14]. BDNF is a neurotrophin that facilitates neurogenesis, neuroprotection, neuroregeneration, cell survival, and synaptic plasticity, as well as formation, retention, and recall of memory in the hippocampus and frontal cortex [15,16]. In rodents, BDNF acts as an anorexigenic factor, its postnatal reduction leads to hyperphagia and obesity [17]. Interestingly, the administration of fructo-oligosaccharides and galacto-oligosaccharides increased hippocampal BDNF and *N*-methyl-d-aspartate receptor subunit (NR1) expression in rodents possibly through the involvement of gut hormones [18].

Glial derived neurotrophic factor (GDNF) is a potent promoter of neuronal survival in the central nervous system and peripheral nervous system. It has been shown to have effect on the number of cell populations including sensory and autonomic ganglia, Purkinje cells of the cerebellum, hippocampal neurons, as well as noradrenergic, serotoninergic, and cholinergic neurons [15]. Several studies have shown that chronic hypothalamic or nigrostriatal expression of GDNF in rodents and primates can induce weight loss in animals with age-related obesity and prevent weight gain in young animals [19,20,21]. Recently, a novel role of GDNF on the regulation of high-fat diet-induced obesity through increased energy expenditure has been noted [22].

On the other hand, oxidative stress is defined as an imbalance between production of oxygen-free radicals and antioxidant defense mechanisms, leading to a cascade of reactions in which lipids, proteins, and DNA may be damaged [23]. A high-fat diet (HFD) consumption led to increased brain oxidative damage through induction of lipid peroxidation in the hippocampus [24,25] and protein carbonyls [26]. Many studies have shown the prebiotic effect of fructans in obesity both in animals and humans [5,6,27,28], though the neurobiological effects of fructans intake have not been explored. To increase neurotrophic factors and reduce oxidative stress in the brain caused by high-fat diet consumption, a dietary supplementation with agavins is proposed. The aim of this work is to investigate the impact on body weight gain occurring with agave fructans supplementation. We focused on the influence of agavins on neurotrophic factors and oxidative stress levels in the brain of obese mice under a high-fat diet.

## 2. Results and Discussion

### 2.1. Agavins Supplementation Protects Against Body Weight Gain and Controls the Food Intake Mechanism from Diet-Induced Obesity

**Figure 1:**
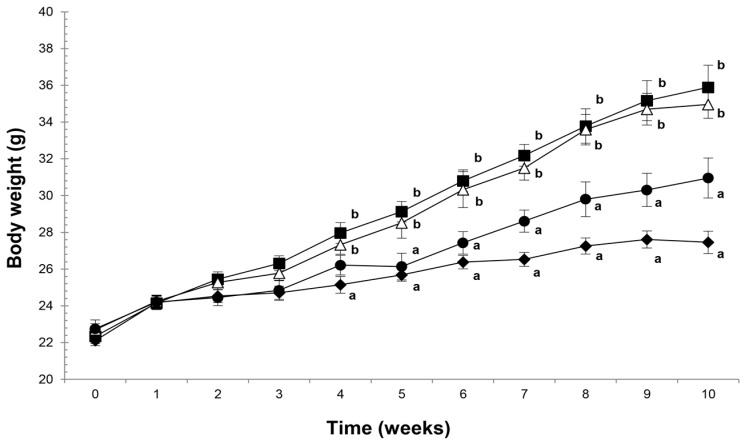
Body weights in mice fed with standard diet (SD, ⧫); high fat diet (HFD, ∎); high fat diet with agave fructans 5% (HFD/A5, ∆); high fat diet with agave fructans 10% (HFD/A10, ●). One-way ANOVA with repeated measures and Tukey contrasts were performed to compare differences among groups. The data are represented as the mean with their standard errors of the mean (SEM). *N* = 10. Mean values with unlike letters (a,b) were significantly different (*p* < 0.05).

Significant differences in the weight of mice were observed between the HFD and HFD/A5 groups when compared to the SD group at the beginning of the 4 weeks of treatment (*p* < 0.05) and at the end of experiment for 10 weeks (*p* < 0.05). However, no difference was seen with the HFD/A10 group (Figure 1).

By analyzing the body weight gain at the end of treatment, it was observed that the group of mice fed a standard diet (SD) had a weight gain of only 5.35 ± 0.19 g, whereas HFD-fed mice (HFD) gained 13.34 ± 0.17 g (*p* < 0.05), more than double. On the other hand, HFD-fed mice a dose of 5% agave fructans (HFD/A5) exhibited a body weight gain similar to the HFD group of 12.26 ± 0.55 g (*p* < 0.05), but not the group HFD-fed a dose of 10% agave fructans (HFD/A10), which only gained 8.2 ± 0.18 g (*p* < 0.05) (Table 1).

According to several reports from animal and human studies, fructan supplementation promotes weight loss in both obese animals and humans [7,29,30,31,32,33,34,35,36,37,38,39] through several mechanisms; one of which is by mediation of SCFAs through regulation of whole-body energy homeostasis [7,40,41,42].

The HFD-fed mice group that increased their intake (59.6 ± 2.48 kJ/day/mice) showed a statistically significant difference (*p <* 0.05) than the group who consumed SD (56.1 ± 2.0 kJ/day/mice). However, only the mice group that consumed HFD and 10% agavins decreased their ingestion (52.6 ± 2.63 kJ/day/mice) significantly compared to the HFD group (*p <* 0.05) (Table 1). At the end of treatment it was observed that the HFD/A5 and HFD/A10 groups reduced their water intake significantly (4.45 ± 0.40; 4.32 ± 0.36 mL/day/mice) even when compared to the SD group (*p <* 0.05) (Table 1).

Regarding to satietogenic effect of fructans, agave fructans reduced food intake and water intake in mice fed with a high-fat diet, which can be explained by the increasing GLP-1 and YY peptide concentrations in plasma of fructan supplementation that contributed to changes on appetite and glucose release responses [7,31,38]; this mechanism is mediated through SCFAs since it improves satiety [43,44]. 

### 2.2. Agavins Reduces Triglycerides but Does Not Modify Glucose or Cholesterol in Mice Fed HFD

Regarding the effect of different sources of fructans on blood metabolic parameters such as glucose, triglycerides and cholesterol, there are several studies that show inconsistent results [6,8,45]. However, in this work we found that agave fructans had an impact on triglycerides levels in mice fed with HFD and supplemented with 5% (7.28 ± 0.28 mM) and 10% (7.04 ± 0.17 mM) agavins (Table 2) suggesting that these have an impact on the synthesis of triglycerides. However, no significant differences were observed in glucose and cholesterol concentrations among experimental groups after 10 weeks of treatment (Table 2).

Several studies have shown that fructans reduced plasma lipid levels in both animals and humans [31,46,47,48,49]; however, no significant differences were observed in glucose or total cholesterol concentrations between all experimental groups after 10 weeks of treatment (Table 2) in this experimental model mouse.

### 2.3. Agavin Fermentation Induced SCFAs Production

**Figure 2:**
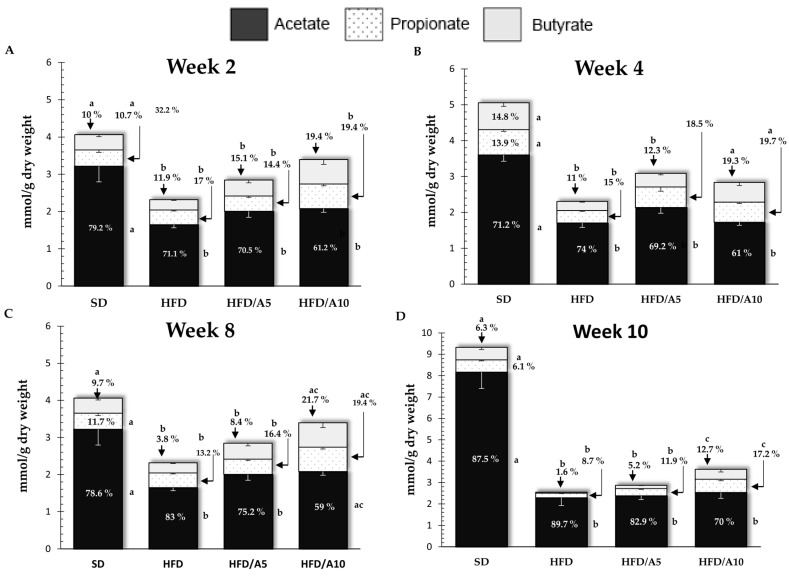
Total fecal mice SCFAs levels at (**A**) 2 weeks, (**B**) 4 weeks, (**C**) 8 weeks and (**D**) 10 weeks. SD, standard diet; HFD, high-fat diet; HFD/A5, high-fat diet supplemented with 5% agavins; HFD/A10, high-fat diet supplemented with 10% agavins. One-way ANOVA and Tukey contrasts were performed to establish differences among groups. The data are represented as the mean with their standard errors of the mean (SEM). *N* = 10. Mean values with unlike letters (a,b,c) were significantly different (*p <* 0.05).

Some physiological effects of fructans depend on the degree to which they may be fermented by gut microbiota, and due to this fact, we decided to evaluate the production of SCFAs generated by the fermentation of agavins. In this study, we found that acetate was the most abundant SCFAs produced when compared to the levels of both propionate and butyrate throughout the treatment duration. We observed that from the 2nd week of treatment, the levels of acetate decreased in HFD (1.64 ± 0.08 mmol/g) with respect to SD (3.21 ± 0.42 mmol/g) (*p <* 0.05) but increased in HFD/A5 (2.00 ± 0.16 mmol/g) and even more in HFD/A10 (2.08 ± 0.09 mmol/g) (*p <* 0.05) as well as the levels of propionate that were also lower in HFD (0.39 ± 0.02 mmol/g) with respect to HFD/A10 (0.65 ± 0.04 mmol/g) that even increased beyond the baseline levels (SD; 0.43 ± 0.06 mmol/g) (*p <* 0.05). In the same way, the levels of butyrate were also lower in the HFD group (0.27 ± 0.01mmol/g) and again higher in HFD/A10 (0.65 ± 0.12 mmol/g) (*p <* 0.05) (Figure 2A). At week 4th, the levels of butyrate remained low for the HFD group (0.25 ± 0.01 mmol/g) with respect to SD (0.74 ± 0.09 mmol/g) and an increase was observed in the HFD/A5 group (0.37 ± 0.04 mmol/g) and HFD/A10 (0.54 ± 0.08 mmol/g) (*p* < 0.05) (Figure 2B). This effect persisted until the 8th (Figure 2C) and 10th weeks only at the levels of propionate and butyrate not for acetate (Figure 2D) (Table 3). According to these results, in overweight mice, agave fructans increased the levels of SCFAs more than oligofructose. These differences on the effect of oligofructose and agave fructans on SCFAs production may be due to the structural differences among these prebiotics [6].

Supplementation with agave fructans primarily increased levels of acetate, propionate and butyrate, in addition to lowering blood triglyceride; according to this process, fermentation of OFS to SCFAs, in particular propionate, decreased the expression and activity of hepatic lipogenic enzymes inhibiting hepatic lipid synthesis [50,51] which can be attributed to the aforementioned supplementation effect. Figure 2 presents the levels and percentages of total SCFAs and significant differences among groups.

### 2.4. Prevention of Decrease in the Levels of BDNF and GDNF Caused by Fat Intake

The bidirectional communication between the brain and the gut in the area of food intake, satiety, and the regulation of the digestive tract [52,53,54] has been heavily studied but the neurobiological benefits of fructan intake have not been explored.

However, what has been explored is that circulating propionate and butyrate produced by gut microbial fermentation can be carried by monocarboxylate transporters abundantly expressed at the blood-brain barrier, and they can be taken for glia and neurons as the main source of energy [55]. In fact, it is well known that fructans increasing SCFAs levels might be contributing to this benefit in the brain.

**Figure 3:**
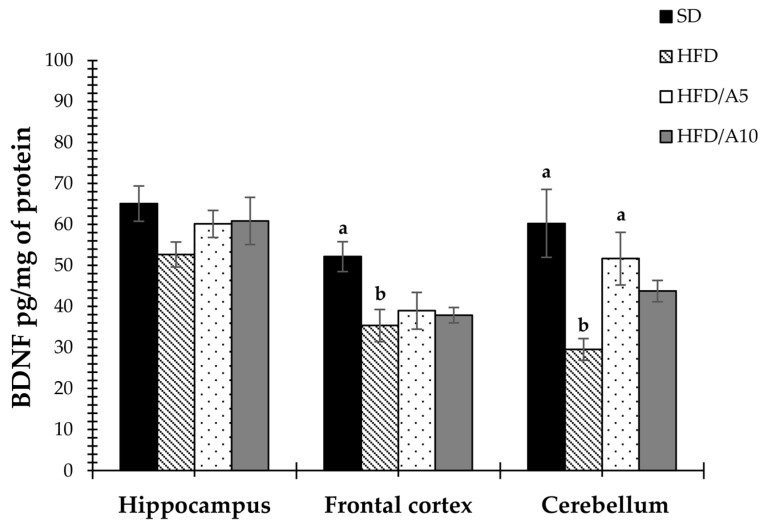
Effects of fructans on brain-derived neurotrophic factor (BDNF) levels in the hippocampus, frontal cortex and cerebellum of mice fed with standard diet (SD); high-fat diet (HFD); high-fat diet with agave fructans 5% (HFD/A5); high-fat diet with agave fructans 10% (HFD/A10). One-way ANOVA and Tukey contrasts were performed to compare differences among groups. The data are represented as the mean with their standard errors of the mean (SEM). *N =* 10. Mean values with unlike letters (a,b) were significantly different (*p <* 0.05).

The levels of neurotrophic factors in the hippocampus, frontal cortex and cerebellum, all areas involved in memory and learning that are affected by the intake of saturated fats [12,13,14], were also determined. As shown in Figure 3, BDNF levels decreased in the frontal cortex and cerebellum of high-fat diet mice (HFD) (35.34 ± 3.91 and 29.53 ± 2.64 pg/mg protein, respectively) as compared with control mice (SD) (52.14 ± 3.63 and 60.23 ± 8.28 pg/mg protein, *p <* 0.05). The agave fructans treatment at 5% was capable of reestablishing BDNF levels in cerebellum of mice fed a high-fat diet (51.65 ± 6.43 pg/mg protein, *p <* 0.05).

The first study in this field was done by Savignac et al. [18] where FOS and GOS were administered to healthy rats resulting in increased levels of BDNF. This is the first report on the agave fructans effect on BDNF levels in the cerebellum of mice fed a high-fat diet. In healthy rodents, prebiotic-mediated proliferation of gut microbiota increases brain BDNF expression [18]. In our study, we are suggesting that this is the correct mechanism by which fructans may prevent cognitive deficits and neurodegenerative diseases resulting from obesity. The possibility that prebiotics alter brain signaling independently of the gut microbiota cannot be ruled out, and a direct interaction between fructans and the gut mucosa has also been shown to influence the response of the immune system [8].

On the other hand, BDNF can act as an anorexigenic factor reducing food intake and body weight in rats [56,57] indicating that this may be another factor influencing weight loss and decreasing caloric intake in mice.

**Figure 4:**
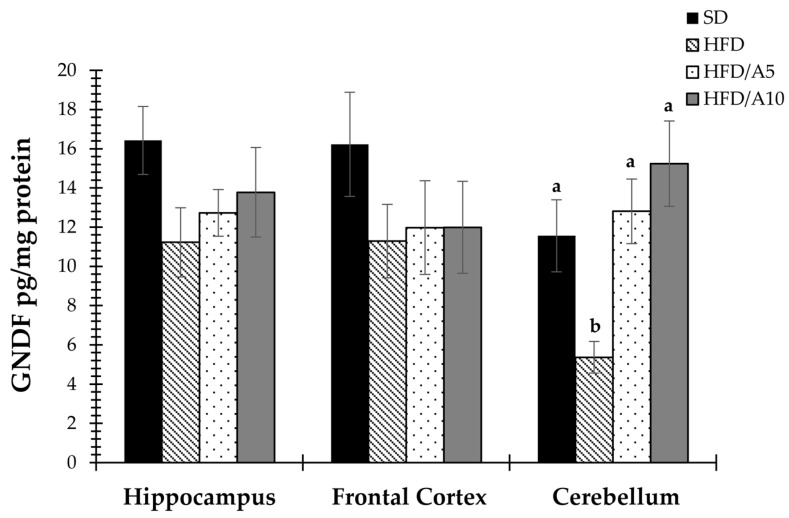
Effects of fructans on glial-derived neurotrophic factor (GDNF) levels in the hippocampus, frontal cortex and cerebellum of mice fed with standard diet (SD); high-fat diet (HFD); high-fat diet with agave fructans 5% (HFD/A5); high-fat diet with agave fructans 10% (HFD/A10). One-way ANOVA and Tukey contrasts were performed to compare differences among groups. The data are represented as the mean with their standard errors of the mean (SEM). *N =* 10. Mean values with unlike letters (a,b) were significantly different (*p <* 0.05).

The GDNF levels in the hippocampus, frontal cortex and cerebellum of all mice groups were also determined (Figure 4). GDNF has been useed as a treatment or a prevention of obesity [22], a novel roll for this neurotrophin. The effect of fructans on GDNF levels has not been previously explored and such information might have therapeutic relevance. As we report here, a treatment with agavins, at least in the cerebellum, restores and even increases the levels of GDNF of HFD/A5 and HFD/A10 (12.80 ± 1.64 and 15.24 ± 2.17 pg/mg protein, *p <* 0.05) by the effect of high-fat diet (HFD) (5.36 ± 0.8 pg/mg protein) as compared to control mice (SD) (11.55 ± 1.83 pg/mg protein, *p <* 0.05) (Figure 4).

Moreover, several studies have shown that chronic hypothalamic or nigrostriatal expression of GDNF in rodents and primates can induce weight loss in animals with age-related obesity and prevent weight gain in young animals [19,20,21]. We have shown that agave fructans reverse the loss of GDNF in cerebellum of obese mice. This is particularly important because a recent study indicates that GDNF has the ability to protect against high-fat diet-induced obesity [22].

Thus, our results suggest that agavins could be an alternative to prevent loss of BDNF and GDNF in the brain and avoid or decrease the development of neurodegenerative diseases resulting from obesity.

### 2.5. Agavins Decreased Lipid Peroxidation in Regions Involved in Memory and Learning

**Figure 5:**
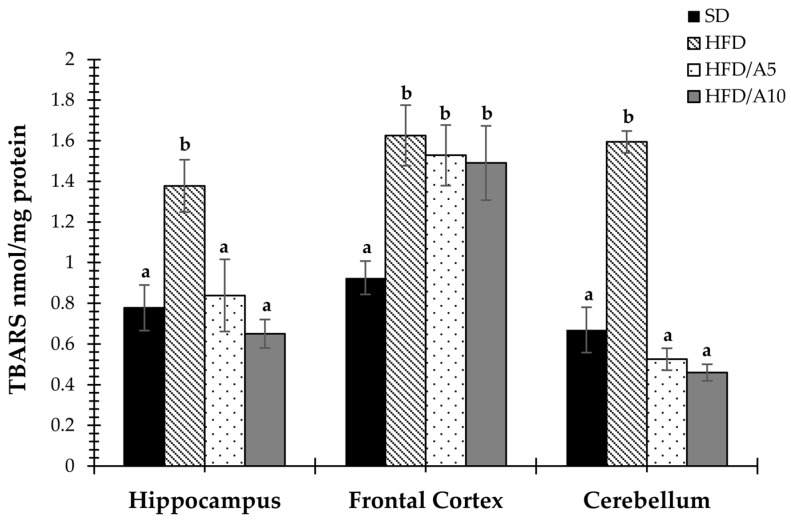
Effects of fructans on TBARS levels in the hippocampus, frontal cortex and cerebellum of mice fed with standard diet (SD); high-fat diet (HFD); high-fat diet with agave fructans 5% (HFD/A5); high-fat diet with agave fructans 10% (HFD/A10). One-way ANOVA and Tukey contrasts were performed to compare differences among groups. The data are represented as the mean with their standard errors of the mean (SEM). *N =* 10. Mean values with unlike letters (a,b) were significantly different (*p <* 0.05).

The effect of agave fructans on oxidative damage indexes through the levels of lipid peroxidation was analyzed (Figure 5). Here, a significant reduction on TBARS levels was observed in obese mice brains. It was found that the levels of TBARS in hippocampus, frontal cortex and cerebellum of HFD mice were elevated (1.37 ± 0.12, 1.62 ± 0.14 and 4.59 ± 0.05 nmol/mg protein, respectively) as compared to the control mice (SD) (0.77 ± 0.11, 0.92 ± 0.08, 0.66 ± 0.11, *p* <0.05, respectively). Interestingly, the antioxidant potential of the supplemented agave fructans was observed in the hippocampus, frontal cortex and cerebellum in both concentration treatment groups HFD/A5 (0.83 ± 0.17, 1.52 ± 0.14 and 0.52 ± 0.05 nmol/mg protein, *p* <0.05) and HFD/A10 (0.65 ± 0.07, 1.49 ± 0.18 and 0.45 ± 0.04 nmol/mg protein, *p <* 0.05) as compared to HFD in all brain regions (Figure 5). There are no studies describing the antioxidant effects in specific regions of the brain implicated in cognitive processes.

### 2.6. Protection against Protein Oxidative Damage in Specific Brain Regions

**Figure 6:**
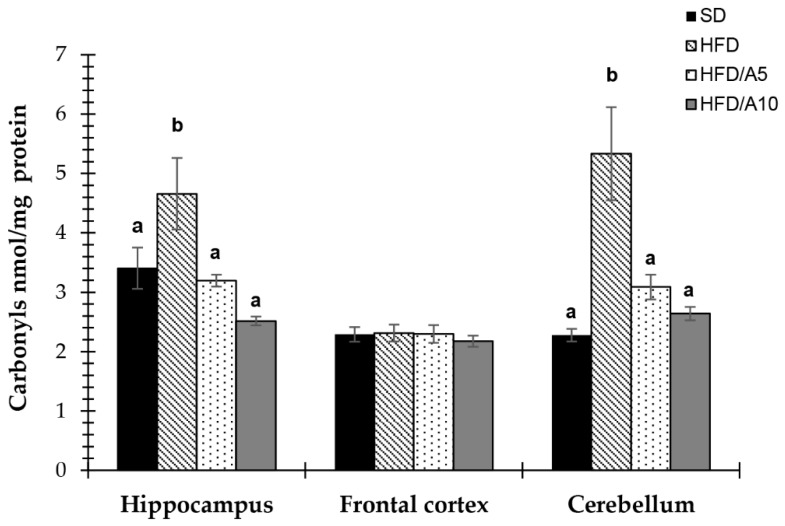
Effects of fructans on protein carbonyls levels in the hippocampus, frontal cortex and cerebellum of mice fed with standard diet (SD); high-fat diet (HFD); high-fat diet with agave fructans 5% (HFD/A5); high-fat diet with agave fructans 10% (HFD/A10). One-way ANOVA and Tukey contrasts were performed to compare differences among groups. The data are represented as the mean with their standard errors of the mean (SEM). *N =* 10. Mean values with unlike letters (a,b) were significantly different (*p <* 0.05).

In the hippocampus, the treatment with agave fructans prevented carbonyls formation in HFD/A5 and HFD/A10 groups (3.19 ± 0.10 and 2.51 ± 0.07 nmol/mg protein) when compared to HFD group (4.65 ± 0.60, *p <* 0.05). Moreover, in the cerebellum, high-fat diet (HFD) increased the levels of protein carbonyls (5.33 ± 0.78 ng/mg protein) as compared with standard diet (SD) (2.27 ± 0.10 ng/mg protein, *p <* 0.05). While treatment with agave fructans at 5% and 10% decreased carbonyls levels (3.08 ± 0.21 and 2.64 ± 0.11, *p <* 0.05, respectively) when compared to HFD (Figure 6).

In the same way, the supplementation with agave fructans prevented the oxidation of proteins in obese mice brain regions. Thus, our results indicate that agave fructans are working as antioxidants in a high-fat diet fed mice model. Regarding this effect, in a very recent study with hypercholesterolemic rats fed with diets containing *Agave tequilana* as dietary fiber and Jamaica calyces, the authors reported that both treatments improved the redox status by reducing the malondialdehyde serum levels and protein oxidative damage in the liver [45]. However, the mechanism remains unclear.

## 3. Experimental

### 3.1. Animals and Diets

Forty male C57BL/6 mice were purchased from UPEAL (Mexico City, Mexico). Mice were housed individually in cages with access to plastic nest-boxes, tunnels and nest building material. Tunnels and nest-boxes were rearranged once a week and replaced with new ones once a week, to promote a stimulating environment. Once a week dirty sawdust was removed and replaced with new sawdust, and the entire cage was replaced for a cleaned cage. Mice were handled as little as possible. 

The mice had a 12-h light/dark cycle and were given free access to diet and water. After a week adaptation period, animals were distributed into 4 experimental groups (*n =* 10). Thus, mice were fed a standard diet (SD) (Picolab rodent diet, 5053, LabDiet^®^, Richmond, IN, USA) (group 1) or a high-fat diet (HFD) (DIO rodent purified diet with 60% energy from fat-blue 58Y1, Test Diet^®^, Richmond, IN, USA) (group 2), and two groups supplemented daily with 5% (HFD/A5) (group 3) or 10% (HFD/A10) (group 4) with agavins (Ingredion, Guadalajara, Mexico) in water (5 or 10 g/100 g of diet, respectively) as previously described [6,33]. Mice were treated during 10 weeks. Food intake, taking into account spillage, was recorded daily for 10 weeks. Water consumption was also recorded daily to analyze prebiotic intake. The mean daily energy intake (kJ/day) was calculated as follows: food intake (g) X energy value of diet (kJ/g). The energy value for the STD diet was 14.26 kJ/g and the HFD was 21.33 kJ/g.

The experimental protocol and all animal procedures were approved and conducted according to the Guidelines of the Institutional Care and Use of Laboratory Animals Committee from SIACUAL system of CINVESTAV-Mexico (Permit Number: 0092-14) in accordance with current Mexican legislations (NOM-062-ZOO-1999).

### 3.2. Measurement of Body Weight Gain and Glucose, Triglyceride and Total Cholesterol Serum Levels

The body weight of each mice was assessed with an analytical balance and recorded every seven days until the end of the experiment. Blood samples were obtained from the caudal vein every 15 days until the end of the experiment and immediately used for the quantitative determination of glucose, triglycerides, and cholesterol (Accutrend, Roche Diagnostics^©^, Mannheim, Germany) with the Accutrend Plus meter (Roche Diagnostics^©^) (according to the manufacturer´s instructions). At the end of the experiment mice were sacrificed by cervical dislocation and the hippocampus, frontal cortex and cerebellum were dissected and stored in 300–500 µL of Buffer A: HEPES 10 mM, pH 7.9, Nonidet P-40 (Sigma-Aldrich, Toluca, Mexico) at 0.6%, NaCl (Fermont, Mexico City) 150 mM, EDTA) 1 mM and complete Mini Protease Inhibitor (Roche Diagnostics^©^); at −20 °C prior to processing.

### 3.3. Quantification of Short-Chain Fatty Acids (SCFAs) in Feces

To individually quantify short-chain fatty acids (SCFAs) we collected mice feces once a week for the whole experiment. SCFAs were measured with an HP 5890 series II gas chromatograph (Hewlett-Packard, Waldbronn, Germany) equipped with an HP-20 M column and a flame ionization detector as described in [6] with a minor modifications. Briefly, 0.2 g of feces were weighed and acidified using 70 µL of H_2_SO_4_ and 480 µL of water and centrifuged at 14,000 rpm for 10 min. SCFAs were extracted by shaking with 0.2 mL of diethyl ether and finally centrifuged at 14,000 rpm for 5 min. One µL of the organic phase was injected directly into the capillary column. The initial temperature was 80 °C and the final temperature was 200 °C, using He and N_2_ as the carrier gases and O_2_ as combustion gas. Samples were analyzed in duplicates. Calibration curves of acetic, propionic and butyric acids were used to carry out SCFAs quantification in the samples.

### 3.4. Tissue Homogenization and Measurement of Protein Concentration

Brain samples were homogenized in a 300–500 µL of buffer A in a silent crusher device (Roche Diagnostics©) at 4°C. Protein concentration of samples was determined using the bicinchoninic acid method as described in Franco-Robles et al. [58]. Briefly, a 1:20 dilution of samples was performed. Bicinchoninic acid (Sigma-Aldrich) (0.2 g) was dissolved into 5 mL of H_2_O to one ELISA plate of 96 wells. Two hundred µL of CuSO_4_ (Fermont, Mexico City, Mexico) (4%) and 5.2 mL of micro reagent A were added. In the wells 0, 2, 4, 6 and 8 µL of BSA were loaded and adjusted to 100 mL with H_2_O. Eight µL of the samples were then loaded in their respective wells, all measurements were done in triplicate. One hundred µL of the working solution were added to each well and incubated at room temperature for 2–4 h and the plate was read at 540 nm. The plate was covered to prevent evaporation.

### 3.5. Measurement of BDNF and GDNF Levels 

In the total homogenate of mice hippocampus, frontal cortex and cerebellum, BDNF and GDNF levels were measured using the commercial enzyme-linked immunosorbent assay (ELISA) according to the manufacturer’s instructions for BDNF (Mouse BDNF ELISA Kit, MyBioSource, San Diego, CA, USA; catalog number MBS355435) and GDNF (GDNF Mouse ELISA Kit, abcam^®^, Cambridge, MA, USA; catalog number ab171178).

### 3.6. Determination of Thiobarbituric Acid Reactive Substances (TBARS) in the Brain

In the total homogenate of mice hippocampus, frontal cortex and cerebellum, the MDA (malondialdehyde)-TBA adduct formed from the reaction of MDA in samples with TBA were quantified with the OxiSelect™ TBARS Assay Kit (MDA Quantitation) (Cell Biolabs, INC., San Diego, CA, USA; catalog number STA-330) according to the manufacturer’s instructions. Five hundred µg of total homogenate were used. Concentrations were expressed as nmol TBARS/mg of protein in brain tissue.

### 3.7. Measurement of Oxidized Protein

In the total homogenate of mice hippocampus, frontal cortex and cerebellum, the quantification of carbonyls content was performed as described by Franco-Robles et al. [58]. Five hundred µg of total homogenate were used. Carbonyl-DNPH Mix 2 was used as standard control (Supelco, Bellefonte, PA, USA). Concentrations were expressed as nmol carbonyls/mg of protein in brain tissue.

### 3.8. Statistical Analysis

Statistical analysis was performed using the software Statistica 8 (StatSoft Inc, Tulsa, OK, USA). Data obtained from mice were analyzed with one-way ANOVA and ANOVA repeated measures followed by Tukey’s tests for the difference between groups. Data are represented as the means ± standard error of the means (SEM). Values were considered statistically significant if *p <* 0.05.

## 4. Conclusions

We have demonstrated that *Agave tequilana* fructans at 10% supplementation for 10 weeks decreased body weight gain caused by high-fat diet in mice. Interestingly, in cerebellum, agavins at both levels of supplementation (5% and 10%) increased BDNF and GDNF significantly. These results suggest that agavins can produce an anorexigenic effect through these neurotrophic factors. Furthermore, high-fat diet caused oxidative damage in the brain by systemically increasing free radicals; however, supplementation with agavins at both 5% and 10% decreased levels of lipid peroxidation and oxidized proteins in the hippocampus and cerebellum. Additional research is required to determine whether agave fructans may be effective for the prevention and/or treatment of oxidative stress that can lead to cognitive deficits in obese humans.

## Figures and Tables

**Table 1:** Body weight gain, intake food and water in all groups of mice.

Parameters	Control Groups		Treatment Groups	
	SD (*n* = 10)	HFD (*n* = 10)	HFD/A5 (*n* = 10)	HFD/A10 (*n* = 10)
Body weight gain (g)	5.35 ± 0.19 ^a^	13.34 ± 0.12 ^b^	12.26 ± 0.18 ^b^	8.20 ± 0.18 ^a^
Food intake (kJ/day/mice)	56.09 ± 2.08	59.54 ± 2.48 ^b^	56.15 ± 2.38 ^b^	52.66 ± 2.63 ^a^
Daily water intake (mL/day/mice)	5.45 ± 0.46 ^a^	4.79 ± 0.41 ^b^	4.45 ± 0.40 ^b^	4.32 ± 0.36 ^b^

The data are represented as the mean with their standard errors of the mean (SEM). SD, standard diet; HFD, high fat diet; HFD/A5, high-fat diet with agave fructans 5%; HFD/A10, high-fat diet with agave fructans 10%. Ten weeks-old C57BL/6 male mice were fed SD or HFD or HFD/A5 or HFD/A10 for 10 weeks. Mean values with unlike letters (a,b) were significantly different (*p <* 0.05).

**Table 2:** Effects of fructans on blood metabolites in all groups of mice in the 10^th^ week.

Blood Metabolites (mM)	Control Groups		Treatment Groups	
	SD (*n =* 10)	HFD (*n =* 10)	HFD/A5 (*n =* 10)	HFD/A10 (*n =* 10)
Glucose	7.50 ± 0.34	8.84 ± 0.44	8.75 ± 0.38	8.72 ± 0.29
Triglycerides	6.77 ± 0.11 ^a^	8.96 ± 0.79 ^b^	7.28 ± 0.28 ^a^	7.04 ± 0.17 ^a^
Total cholesterol	9.10 ± 0.07	9.22 ± 0.06	9.24 ± 0.04	9.26 ± 0.07

The data are represented as the mean with their standard errors of the mean (SEM). SD, standard diet; HFD, high-fat diet; HFD/A5, high-fat diet with agave fructans 5%; HFD/A10, high-fat diet with agave fructans 10%. Mean values with unlike letters (a,b) were significantly different (*p <* 0.05).

**Table 3:** Short chain fatty acids profiles at 10th week of treatment.

Group/Week	1	2	3	4	5	6	7	8	9	10
Acetic acid (mmol/g)										
SD	2.50 ± 0.24	3.21 ± 0.42 ^a^	4.48 ± 0.76 ^a^	3.60 ± 0.17 ^a^	3.24 ± 0.36 ^a^	4.14 ± 0.60 ^a^	2.90 ± 0.27 ^a^	4.37 ± 0.46 ^a^	3.72 ± 0.59 ^a^	8.16 ± 2.42 ^a^
HFD	1.85 ± 0.18 ^a^	1.64 ± 0.08 ^b^	1.36 ± 0.10 ^b^	1.70 ± 0.12 ^b^	0.81 ± 0.05 ^b^	0.90 ± 0.07 ^b^	1.48 ± 0.24 ^b^	0.90 ± 0.07 ^b^	1.59 ± 0.09 ^b^	2.28 ± 0.36 ^b^
HFD/A5	2.70 ± 0.26	2.00 ± 0.16 ^b^	1.89 ± 0.15 ^b^	2.13 ± 0.16 ^b^	1.25 ± 0.07 ^b^	1.91 ± 0.12 ^b^	1.13 ± 0.09 ^b^	1.43 ± 0.04 ^b^	2.33 ± 0.18 ^b^	2.37 ± 0.17 ^b^
HFD/A10	3.15 ± 0.38 ^b^	2.08 ± 0.09 ^b^	2.12 ± 0.16 ^b^	1.72 ± 0.08 ^b^	1.43 ± 0.08 ^b^	2.62 ± 0.39	1.16 ± 0.12 ^b^	2.20 ± 0.19 ^a,c^	2.68 ± 0.17	2.53 ± 0.27 ^b^
Propionionic acid (mmol/g)										
SD	0.41 ± 0.04	0.43 ± 0.06 ^a^	0.90 ± 0.15 ^a^	0.70 ± 0.04 ^a^	0.79 ± 0.18 ^a^	0.75 ± 0.12 ^a^	0.48 ± 0.04	0.65 ± 0.07 ^a^	1.79 ± 0.25 ^a^	0.57 ± 0.04 ^a^
HFD	0.40 ± 0.02	0.39 ±0.02 ^a^	0.30 ± 0.01 ^b^	0.34 ± 0.02 ^b^	0.13 ± 0.02 ^b^	0.13 ± 0.01 ^b^	0.32 ± 0.04	0.14 ± 0.01 ^b^	0.35 ± 0.13 ^b^	0.22 ± 0.02 ^b^
HFD/A5	0.37 ± 0.02 ^a^	0.40 ± 0.03 ^b^	0.44 ± 0.04 ^b^	0.57 ± 0.11	0.26 ± 0.02 ^b^	0.47 ± 0.16 ^b^	0.27 ± 0.01	0.31 ± 0.03 ^b^	0.58 ± 0.09 ^b^	0.34 ± 0.04 ^b^
HFD/A10	0.68 ± 0.12 ^b^	0.65± 0.04 ^c^	0.76 ± 0.06 ^a^	0.55 ± 0.03	0.69 ± 0.09	0.71 ± 0.22 ^a,c^	0.31 ± 0.08	0.72 ± 0.11 ^a,c^	1.09 ± 0.14 ^c^	0.62 ± 0.07 ^a,c^
Butyric acid (mmol/g)										
SD	0.37 ± 0.05	0.40 ± 0.05	1.10 ± 0.40	0.74 ± 0.09 ^a^	0.88 ± 0.22 ^a^	0.52 ± 0.07 ^a^	0.52 ± 0.09 ^a^	0.53 ± 0.07 ^a^	2.25 ± 0.30 ^a^	0.58 ± 0.10 ^a^
HFD	0.42 ± 0.06	0.27 ± 0.01 ^a^	0.35 ± 0.13	0.25 ± 0.01 ^b^	0.02 ± 0.004 ^b^	0.04 ± 0.003 ^b^	0.15 ± 0.02 ^b^	0.04 ± 0.004 ^b^	0.14 ± 0.04 ^b^	0.04 ± 0.006 ^b^
HFD/A5	0.33 ± 0.04	0.43 ± 0.07	0.41 ± 0.05	0.37 ± 0.04 ^b,c^	0.21 ± 0.02 ^b^	0.26 ± 0.05 ^a,b^	0.12 ± 0.01 ^b^	0.16 ± 0.02 ^b^	0.43 ± 0.11 ^b^	0.15 ± 0.01 ^b^
HFD/A10	0.61 ± 0.15	0.65 ± 0.12 ^b^	0.60 ± 0.08	0.54 ± 0.08 ^a^	0.68 ± 0.17 ^c^	0.86 ± 0.16 ^c^	0.24 ± 0.08 ^b^	0.81 ± 0.16 ^a,c^	1.11 ± 0.19 ^c^	0.45 ± 0.12 ^a,c^

Mice fed with standard diet (SD); high fat diet (HFD); high-fat diet with agave fructans 5% (HFD/A5); high-fat diet with agave fructans 10% (HFD/A10). One-way ANOVA and Tukey contrasts were performed to compare differences among groups. The data are represented as the mean with their standard errors of the mean (SEM). *N* = 10. Mean values with unlike letters (^a,b,c^) were significantly different between groups (*p* < 0.05).
